# ADAR1 Suppresses the Activation of Cytosolic RNA-Sensing Signaling Pathways to Protect the Liver from Ischemia/Reperfusion Injury

**DOI:** 10.1038/srep20248

**Published:** 2016-02-01

**Authors:** Hui Wang, Guoliang Wang, Liyong Zhang, Junbin Zhang, Jinxiang Zhang, Qingde Wang, Timothy R. Billiar

**Affiliations:** 1 Department of Medical Genetics, School of Basic Medicine, Tongji Medical College, Huazhong University of Science and Technology, Wuhan, 430030, China; 2 F1281 UPMC Presbyterian Hospital, 200 Lothrop St, Department of Surgery, University of Pittsburgh Medical Center, Pittsburgh, Pennsylvania, 15261, USA; 3 Department of Emergency Surgery, Union Hospital, Tongji Medical College, Huazhong University of Science and Technology, 1277 Jiefang Avenue, Wuhan, 430022, China; 4 Third Xiangya Hospital, Central South University, Changsha, China 410083

## Abstract

Excessive inflammation resulting from activation of the innate immune system significantly contributes to ischemia/reperfusion injury (IRI). Inflammatory reactions in both IRI and infections share the same signaling pathways evoked by danger/pathogen associated molecular pattern molecules. The cytosolic retinoid-inducible gene I(RIG-I)-like RNA receptor (RLR) RNA sensing pathway mediates type I IFN production during viral infection and the sensing of viral RNA is regulated by adenosine deaminase acting on RNA 1 (ADAR1). Using a model of liver IRI, we provide evidence that ADAR1 also regulates cytosolic RNA-sensing pathways in the setting of ischemic stress. Suppression of ADAR1 significantly enhanced inflammation and liver damage following IRI, which was accompanied by significant increases in type I IFN through cytosolic RNA-sensing pathways. In addition, knocking ADAR1 down in hepatocytes exaggerates inflammatory signaling to dsRNA or endotoxin and results in over production of type I IFN, which could be abolished by the interruption of RIG-I. Therefore, we identified a novel ADAR1-dependent protective contribution through which hepatocytes guard against aberrant cytosolic RLR-RNA-sensing pathway mediated inflammatory reaction in response to acute liver IR. ADAR1 protects against over activation of viral RNA-sensing pathways in non-infectious tissue stress.

Ischemia/reperfusion (IR) is a major cause of liver damage during liver transplantation, hepatic resection, severe trauma, and hemorrhagic shock^1^. Excessive inflammation incurred by activation of the innate immune system plays a pivotal role in the initiation and progression of IR^2^, which shares some common pathways with infections caused by invading pathogens. The innate immune system has been shown to recognize danger-associated molecular pattern molecules(DAMPs) via cell-surface/endosomal pattern recognition receptors (PRRs)^1^,^3^,^4^. The function of membrane-bound PRRs such as TLR4 (Toll like receptor 4) has been widely studied in IR, however, the means by which cytoplasmic PRRs perceive intracellular stress signals and initiate inflammatory cytokine production has not been well studied during this non-infectious condition.

Recently, the cytosolic protein retinoic acid-inducible gene 1(RIG-I) like RNA receptors (RLRs) have been reported to function as a cytoplasm PRR to sense intracellular dsRNA. RLR have been shown to elicit type-І IFN in a cytoplasmic regulatory factor (IRF) dependent manner to trigger an antiviral-response. Meanwhile, Adenosine deaminase acting on RNA (ADAR1), was recently identified to be an suppressor of cytosolic immune responses. ADAR1 is an RNA-binding and editing protein, previously documented to change RNA sequences by converting adenosine to inosine^5^ through its RNA-editing activity. More importantly, we and others determined that ADAR1 plays an anti-inflammatory role by suppressing cytosolic innate immune signaling through its RNA-binding function under conditions simulating viral infection^6^,^7^. Particularly, We also demonstrated that ADAR1 plays essential roles in maintaining liver integrity and normal function and prevents cell death under stress conditions^8^. Based on the observations that, liver IR injury and infections share many of the same innate immune signaling pathways^9^,^10^,^11^,^12^,^13^, and that ADAR1 plays a role in the regulation of innate immune responses^6^,^7^ we hypothesized that ADAR1 would regulate immune responses mediated by RIG-I during IR. In this report, we first investigated ADAR1 expression after liver IR and show that ADAR1 is rapidly upregulated by ischemic stress in the liver. Subsequently, we adjusted ADAR1 expression in liver tissue or hepatocytes which were subjected to a variety of stress conditions and found that suppression of ADAR1 expression leads to increased RIG-I dependent type I IFN production, exaggerated inflammation, and greater organ damage following liver IR. Thus, ADAR1 guards against RIG-I activation in the setting of non-infectious tissue stress.

## Results and Discussion

### ADAR1 protects hepatocytes from damage caused by hypoxia and reoxygenation

**Figure 1:**
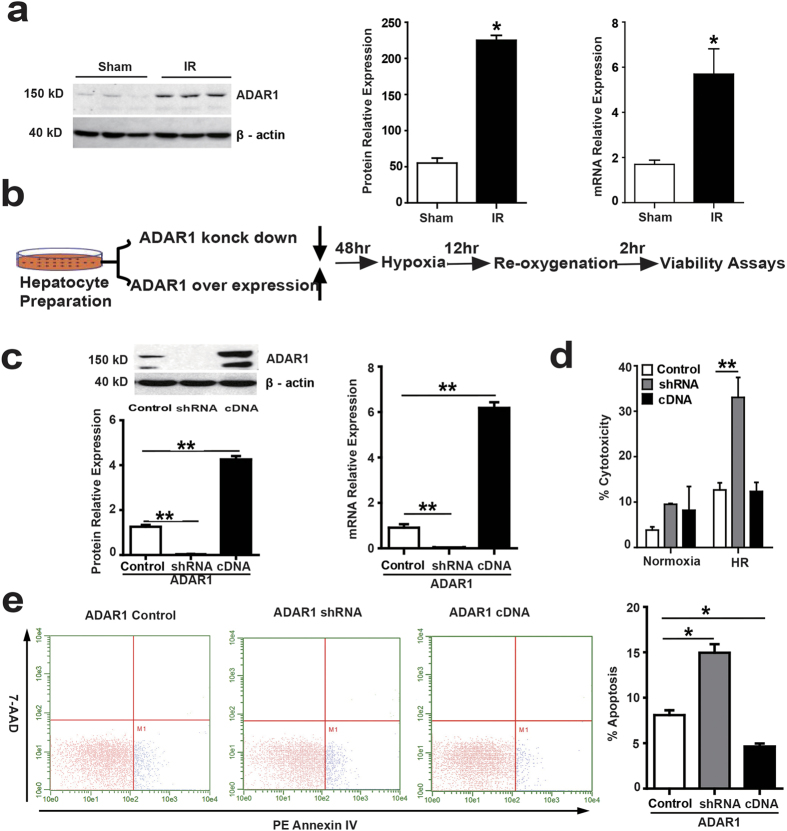
ADAR1 protects hepatocytes from damage caused by hypoxia and reoxygenation. (**a**) The expression of ADAR1 protein and mRNA was measured with western blotting and real-time PCR in the ischemic liver lobes of C57BL/6 mice after 6 hours reperfusion following 1 hour ischemia/reperfusion (IR) (n = 5). (**b**) Schematic diagram of testing the essential role of ADAR1 in hepatocytes under hypoxia and reoxygenation (H/R). The isolated primary hepatocytes were transfected with adenovirus containing ADAR1-specific shRNA or ADAR1 cDNA for the purpose of knocking down or over expressing ADAR1. The hepatocytes were then cultured under 12 hours of hypoxia and 2 hours of reoxygenation and cellular viability and cytokines were detected. (**c**) Knockdown or overexpression of ADAR1 was identified using western blotting and real-time PCR. Control represents primary hepatocytes transfected with vehicle adenovirus (n = 5). shRNA represents hepatocytes transfected with adenovirus containing ADAR1-specific shRNA (n = 5), cDNA represents hepatocytes transfected with adenovirus containing ADAR1 cDNA (n = 5). (**d**) Estimation of hepatocyte damage by detection of LDH released into the media under HR conditions (n = 5). (**e**) Analysis of hepatocyte apoptosis by flow cytometry after staining cells with Annexin V (n = 5) (* p ≤ 0.05; **p ≤ 0.01).

Although ADAR1 plays a critical role in liver development^8^,^14^ and regulates viral cytosolic RNA-sensing pathways in innate immune responses^6^,^15^, the function of ADAR1 in acute non-infectious inflammation resulting from ischemic tissue stress has not yet been investigated. To determine the role of ADAR1 in IR-induced liver damage, we first examined changes in ADAR1 expression during liver IR. Both ADAR1 protein and mRNA levels were significantly increased in the liver at 6 hours of reperfusion following 60 minutes of warm partial liver ischemia (Fig. 1a).

We next examined whether the increased ADAR1 expression was the cause or result of the injury. The lethality of the ADAR1 knockout^8^,^14^ and the severe disruption of liver structure even in conditional ADAR1 liver knockout mice^8^ prevented us from studying the function of ADAR1 in liver IR using knockout mice. Hepatocytes constitute most of the liver mass and undergo damage during IR. Therefore, we initially examined the effect of ADAR1 in *in vitro* cultured primary hepatocytes utilizing ADAR1 overexpression and knockdown strategies by using adenoviruses expressing ADAR1 cDNA or ADAR1-specific shRNA, respectively (Fig. 1b). The modulation of ADAR1 expression was confirmed by western blot and real-time polymerase chain reaction (PCR) (Fig. 1c). First, we determined whether the modulation of ADAR1 expression alters cell death in cultured primary hepatocytes exposed to hypoxia and re-oxygenation (H/R). A dramatic increase in cell death, as measured by LDH release, was observed following H/R when ADAR1 expression was suppressed (Fig. 1d). No change in LDH levels was seen in normoxic conditions following either ADAR1 suppression or overexpression. Apoptosis, as detected by Annexin V staining and flow cytometry analysis, was significantly increased in ADAR1 knockdown hepatocytes (Fig. 1e). In contrast, overexpression of ADAR1 suppressed apoptosis under these conditions.

### ADAR1 protects the liver from IRI in mice

**Figure 2:**
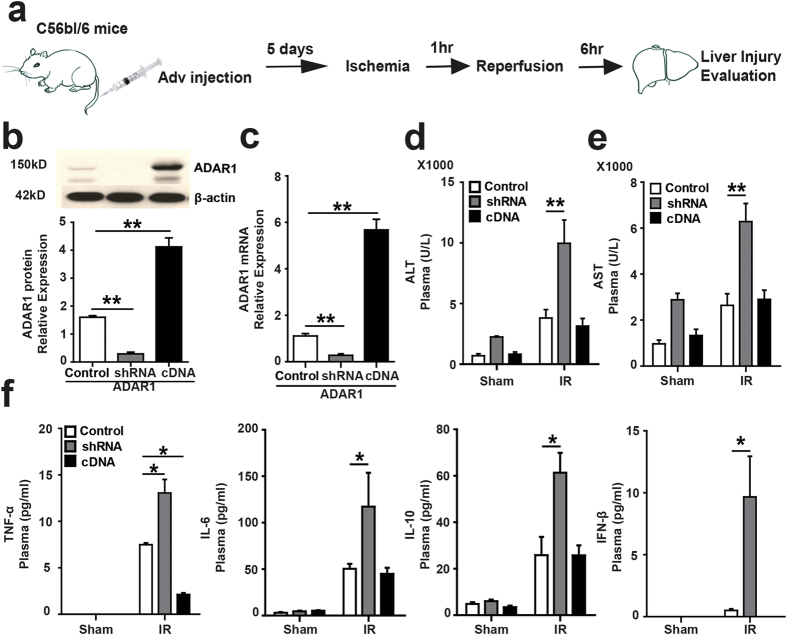
Knockdown ADAR1 aggravates liver function and some cytokines release after liver IR. (**a**) Schematic diagram for exploring the role of ADAR1 in liver IR *in vivo*, drawing by Hui Wang. (**b**) ADAR1 expression in liver lobes was confirmed using western blotting and (**c**) real-time PCR via tail injections of adenovirus containing ADAR1-specific shRNA or ADAR1 cDNA, respectively. Control represents liver lobes transfected with vehicle adenovirus (n = 7), shRNA represents liver lobes transfected with adenovirus containing ADAR1-specific shRNA (n = 7), cDNA represents liver lobes transfected with adenovirus containing ADAR1 cDNA (n = 7). (**d**) The plasma alanine aminotransferase (ALT) and (**e**) aspartate transaminase (AST) levels were analyzed to estimate liver injury (n = 7). (**f**) TNF-α, IL-6, IL-10, and IFN-β levels in the medium were measured by ELISA at 6 hours reperfusion following ischemia. (n = 7).(*p ≤ 0.05; **p ≤ 0.01).

We next examined whether ADAR1 exhibits a protective role *in vivo* following liver IR. This experimental sequence is depicted in Fig. 2a. Efficient knockdown or overexpression of ADAR1 protein levels in the liver by ADAR1 shRNA or cDNA expressed from adenoviral vectors were confirmed by western blot or real-time PCR(Fig. 2b,c). Plasma alanine aminotransferase (ALT) and aspartate transaminase (AST) levels were measured to assess liver damage. As shown in Fig. 2d,e, knockdown of ADAR1 significantly increased ALT and AST plasma levels following IR, a finding which is consistent with the results obtained *in vitro* (Fig. 1d). Overexpression of ADAR1 did not suppress ALT or AST levels.

The sterile inflammatory response plays a critical role in the tissue injury induced by IR and we have shown that ADAR1 regulates immune responses to cellular RNAs^6^. Both of these facts prompted us to investigate the possibility that ADAR1 regulates inflammatory signaling during liver IR since abrupt alterations of cytosolic RNA metabolism likely occurs in IR-stressed hepatocytes. Levels of the inflammatory cytokines TNF-α, IL-6, IL-10, and IFN-β dramatically increased in the circulation of ADAR1-knockdown mice following liver IR, while ADAR1 overexpression suppressed TNF-α levels significantly (Fig. 2f). Thus, ADAR1 selectively suppresses inflammatory signaling during liver IR.

**Figure 3:**
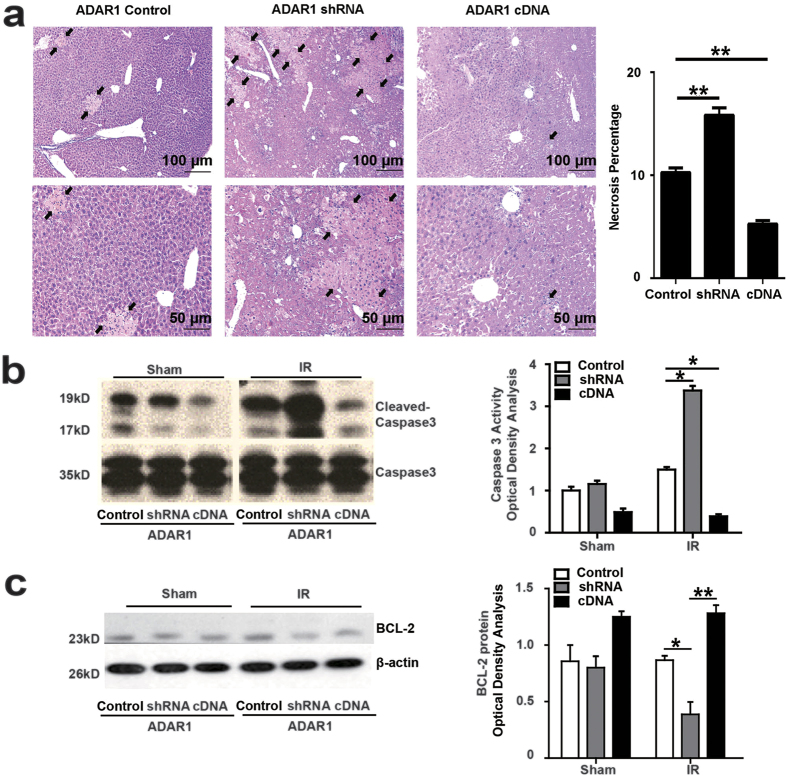
ADAR1 ameliorates necrosis and apoptosis of liver from IRI in mice. (**a**) H&E staining of ischemic liver lobes to show percentage of necrosis in liver sections (n = 7). Small piecemeal necrosis can be seen in control liver; Bridging necrosis and widespread spotty necrosis are revealed in ADAR1 downregulation liver; plasminic loosy hepatocytes with penetration of inflammatory cells is observed in ADAR1 upregulation liver. (**b**) Analysis of caspase3 activity by western blotting (n = 7). (**c**) Level of anti-apoptotic BCL-2 measured by western blotting at 6 hours of liver reperfusion following 1 hour of ischemia (n = 4).(* p ≤ 0.05; **p ≤ 0.01;).

In addition, histologic analysis revealed an obvious increase in liver injury with ADAR1 suppression and less damage when ADAR1 expression was enhanced (Fig. 3a). Comparing with control mice, in which ischemia liver lobes reveal small piecemeal necrosis, notable disruption in hepatic architecture was evident in ADAR1-knockdown mice, including bridging necrosis and widespread spotty necrosis, pycnotic or disintegration of nuclei. Upregulation of ADAR1 weaken liver damage with only scattered spotty necrosis and plasminic loosy hepatocytes, accompanied with penetration of inflammatory cells. Hepatic levels of cleaved caspase 3 also inversely correlated with ADAR1 expression (Fig. 3b), while levels of the anti-apoptotic protein BCL-2 exhibited the opposite pattern (Fig. 3c). Thus, ADAR1 appears to protect the liver from cell death in the setting of IR. However, overexpression appears to specifically suppress apoptosis.

### ADAR1 suppresses type I IFN production in stressed hepatocytes

**Figure 4:**
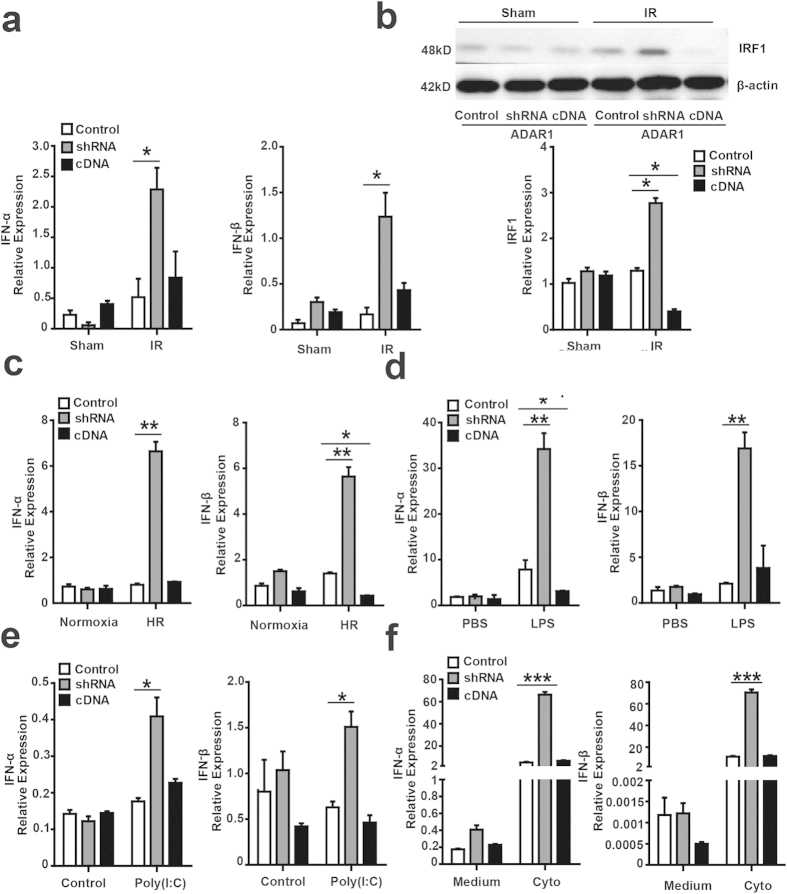
ADAR1 suppresses type I IFN production in stressed hepatocytes. (**a**) Levels of both IFN-α and IFN-β in liver tissues were significantly increased in ADAR1 knockdown mice subjected to IR (n = 4). (**b**) The level of IRF1 protein in liver tissues was increased in ADAR1 knockdown mice but decreased significantly in ADAR1 overexpression mice following IR (n = 4). (**c**) The ADAR1 knockdown hepatocytes produced higher levels of IFN-α and IFN-β upon 2 hours reoxygenation following 12 hours hypoxia. The ADAR1 overexpression hepatocytes released much less IFN-β following hypoxia and reoxygenation (H/R) (n = 4). (**d**) The levels of IFN-α and IFN-β were significantly increased in ADAR1 knockdown hepatocytes after 100 ng LPS treatment for 1 hour and the IFN-α was also decreased in ADAR1-overexpressing hepatocytes(n = 4). (**e**) The levels of IFN-α and IFN-β were slightly but significantly increased in ADAR1 knockdown hepatocytes after 25 μmol poly (I:C) extra-cellular stimulation for 8 hours in medium (n = 4). (**f**) The levels of IFN-α and IFN-β were increased dramatically in ADAR1 knockdown hepatocytes after poly (I:C) was introduced into the cytoplasm; the increase of type I IFN induced by poly (I:C) in the cytoplasm was much more pronounced than poly (I:C) in the medium (n = 4) (*p ≤ 0.05; **p ≤ 0.01; ***p ≤ 0.001).

The IFN pathway is a critical component in liver IR^16^,^17^,^18^. As pro-inflammatory cytokines, IFNs can activate and/or enhance other inflammatory pathways by attracting inflammatory cells and stimulating inflammatory cells to produce more cytokines^19^. We recently demonstrated that ADAR1 suppresses the production of type I IFN in response to viral infection and endogenous RNA stimulation^6^. Therefore, we hypothesized that ADAR1 functions to prevent type I IFN production during liver IR. Real-time-PCR revealed that IFN-α and IFN-β RNA expression were significantly increased in ADAR1-knockdown livers. This was associated with high circulating IFN-α and IFN-β mRNA levels (Fig. 4a) and plasma levels (Fig. 2f) following IR. Interestingly, the expression of IRF1, an IFN-inducible gene that plays a critical role in liver IR^12^, was also significantly increased in ADAR1-knockdown and suppressed with ADAR1 over-expression, respectively (Fig. 4b). Upon IR stress, liver cells produce IFN-β, which stimulates IRF1 expression and causes further inflammatory reactions. Our findings showing that ADAR1 inhibits IFN-β expression in stressed liver combined with previous work showing that type I IFN induces ADAR1 expression^20^,^21^, suggests a regulatory loop to prevent excessive type I IFN production during ischemic stress.

We then explored whether this regulatory relationship extended beyond ischemic stress using cultured primary hepatocytes. We confirmed that ADAR1 knockdown in hepatocytes exposed to H/R resulted in significantly higher type I IFN production, while the overexpression of ADAR1 suppressed IFN-β expression (Fig. 4c). Hepatocytes are often exposed to lipopolysaccharide (LPS) and are the primary site for the clearance of LPS from the circulation^22^. Following treatment with LPS (100 ng/ml), we found a significant upregulation of type I IFN expression in ADAR1 knockdown hepatocytes and suppression of type I IFN by ADAR1 overexpression (Fig. 4d). Hepatocyte stress can also be caused by exposure to nucleic acids released from damaged cells or infected viruses. We recently found that type I IFN expression was suppressed by ADAR1 stimulated by synthesized RNA poly (I:C) in fibroblasts and human cell lines. Then we tested whether ADAR1 also suppresses hepatocyte type I IFN production when exposed to poly (I:C) via the extracellular or intracellular route. At baseline ADAR1 levels or following overexpression of ADAR1, extracellular poly I:C did not stimulate hepatocytes to release type I IFN. In contrast, an increase in type I IFN production was observed when ADAR1 expression was suppressed (Fig. 4e). When poly I:C was introduced directly into the cytoplasm by GeneJammer transfection, a significant increase in type I IFN was observed at baseline levels of ADAR1 expression (Fig. 4f). The induction of IFN-α and IFN-β was further increased by ADAR1 suppression, but not overexpression. These data are consistent with our previous findings in mouse fibroblasts and human cells and indicate that ADAR1 preferentially interacts with cytoplasmic RNA-signaling pathways rather than endosomal TLR pathways^6^. Interestingly, suppression of ADAR1 lead to exaggerated expression of type I IFN expression in cells exposed to ischemic stress. Overexpression of ADAR1 did not show significant suppression of type I IFN expression in hepatocytes, indicating that ADAR1 levels are typically maintained to meet this important function^8^.

### ADAR1 interacts with cytosolic RNA-sensing pathways in hepatocytes

**Figure 5:**
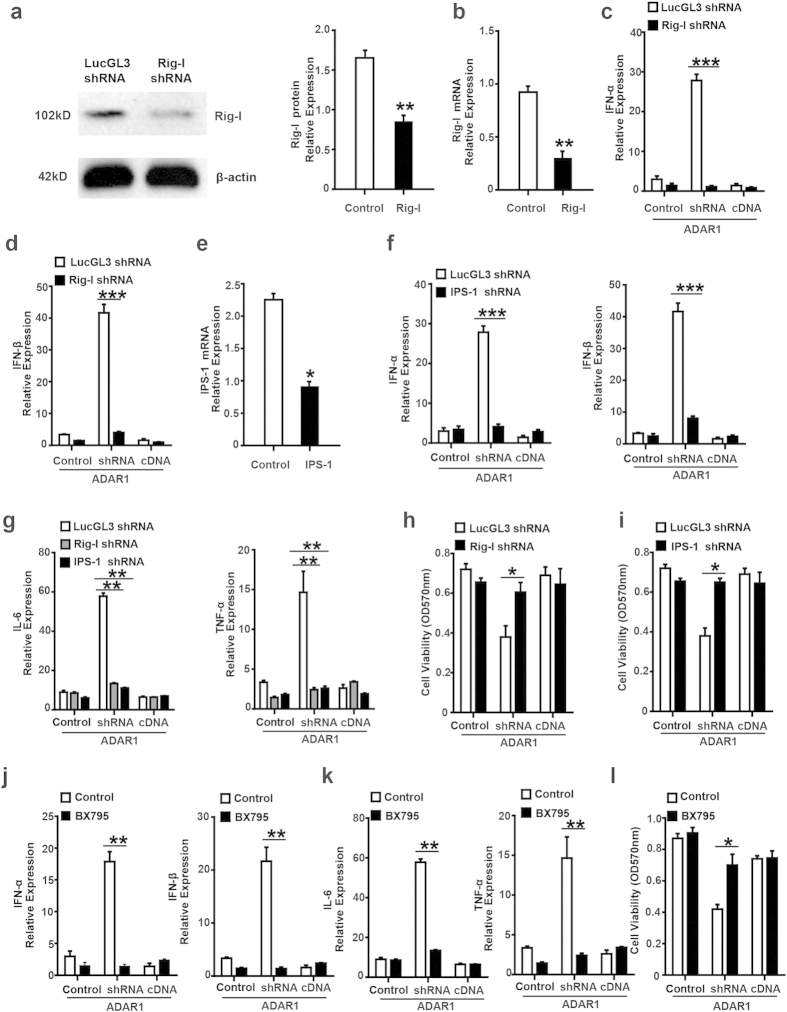
A DAR1 interacts with cytosolic RNA-sensing pathways in hepatocytes. (**a**) Downregulation of RIG-I protein and (**b**) RIG-I mRNA expression were confirmed by western blotting and real time-PCR in hepatocytes transfected by RIG-I shRNA plasmid (n = 4). (**c**) IFN-α and (**d**) IFN-β expression induced by ADAR1 knockdown during hypoxia and reoxygenation(H/R) were completely inhibited by RIG-I knockdown under H/R conditions (n = 4). (**e**) Downregulation of IPS-1 mRNA was confirmed by real-time PCR after hepatocytes were transfected with IPS-1-specific shRNA (n = 4). (**f**) IFN-α and IFN-β production were significantly suppressed by IPS-1 knockdown in ADAR1 knockdown hepatocytes after H/R (n = 4). (**g**) The increase of IL-6 and TNF-α mRNA in ADAR1 knockdown hepatocytes induced by H/R was completely prevented in either RIG-I or IPS-1 knockdown cells (n = 4). (**h**) Cell viability measured by MTT assay showed that cell damage caused by H/R was reduced by either RIG-I knockdown or (**I**) IPS-1 knockdown in ADAR1 knockdown hepatocytes (n = 4). (**J**) BX795, a potent inhibitor of TBK1 and IKK-ε, prevented type I IFN production caused by H/R in ADAR1 knockdown hepatocytes after the drug was administered for 6 hours in advance (n = 4). (**K**) IL-6 and TNF-α mRNA increases in ADAR1 knockdown hepatocytes during H/R were suppressed markedly after BX795 treatment (n = 4). (**L**) Hepatocyte viability was reversed after BX795 treatment in ADAR1 knockdown hepatocytes during H/R. (n = 4)(*p ≤ 0.05; **p ≤ 0.01; ***p ≤ 0.001).

Although components of the IFN pathway, including type I IFNs, IFN receptors, and IRFs, are known to be major contributors in liver IRI, how IR leads to type I IFN expression has not been delineated. Cytosolic RNA-sensing signaling pathways are essential for type I IFN production in viral-infected cells^23^. RLRs are cytosolic RNA receptors that detect cytoplasmic viral RNA^20^,^24^. Upon binding to RNA, RLRs undergo ubiquitination and conformational change, enabling interaction with the mitochondrial adaptor IFN-β promoter stimulator 1 (IPS-1) through their N-terminal caspase recruitment domains^25^. Then, RLRs/IPS-1 relay the activation signal to IRFs and the NF-κB pathway to induce IFN transcription through Tank Binding Kinase-1 (TBK1)/inhibitor of nuclear factor kappa-B kinase-ε(IKK-ε)^19^. If IFN production in ADAR1-knockdown hepatocytes occurs through this cytosolic RNA-sensing pathway, the inhibition of these signal relay cascades should reduce the induction of type I IFN production by ADAR1 knockdown in stressed hepatocytes. To test this hypothesis, we first interrupted cytosolic RNA sensing by knocking down RIG-I with siRNA treatment. RIG-I suppression was confirmed by western blot and quantitative real-time PCR (Fig. 5a,b). As before, knocking ADAR1 down caused a dramatic increase in IFN-α and IFN-β expression in hepatocytes following H/R, which was prevented by RIG-I knockdown at the same time. Knocking RIG-I down even synergized with the effect of ADAR1 overexpression and further suppressed type I IFN expression in the hepatocytes following H/R. (Fig. 5c,d).We also examined whether the suppression of molecules downstream of RLR could negate the effects of ADAR1 suppression on IFN production. We knocked down IPS-1 in hepatocytes using specific siRNA (Fig. 5e). Similar to knockdown of RIG-1, knocking IPS-1 down abolished the increase in type I IFN production caused byADAR1 knockdown during H/R (Fig. 5f). Furthermore, we observed significant increases in the inflammatory cytokines IL-6 and TNF-α when ADAR1 was down-regulated, which was inhibited by knockdown of RIG-I or IPS-1 at the same time (Fig. 5g). Cell viability, as measured by MTT assays, was enhanced by the knockdown of RIG-I or IPS-1in ADAR1-knockdown hepatocytes (Fig. 5h,i).These data indicate that ADAR1 suppresses aberrant cytosolic RNA-sensing pathways in hepatocytes to prevent type I IFN production during hypoxic cell stress. Thus far, our data demonstrates that in H/R-treated hepatocytes, ADAR1 suppresses type I IFN production through inhibition of the cytosolic RNA-sensing pathway.

Next, we investigated the potential of this cytosolic RNA-signaling pathway as a drug target to prevent or reduce hepatocyte damage due to H/R stress. BX795, an amino pyrimidine compound, is a potent inhibitor of TBK1 and IKK-ε^26^,^27^ TBK1/IKK-ε are critical kinases in the cytosolic RNA-signaling pathway. These kinases phosphorylate the transcription factors IRF3/7 and Iκ-B to activate inflammatory cytokine expression. As shown in (Fig. 5j), BX795 completely suppressed the exaggerated type I IFN production caused by ADAR1 knockdown in the setting of H/R. This was also associated with suppression of IL-6 and TNF-α production (Fig. 5k). As expected cell damage induced by ADAR1 knockdown in response to H/R was prevented by BX795 (Fig. 5l). This result further confirmed that the RIG-I cytosolic RNA-sensing pathway was underlying the exaggerated type I IFN production in the IR-stressed liver when ADAR1 was downregulated. These findings also indicate that inhibitors for this signaling pathway, such as BX795, could be a potential drug candidate to protect the liver from IRI.

The RLR-initiated cytosolic RNA-sensing signaling pathway is well-known for its essential role in the innate immune response that defends against viral infection through inducing type I IFN production^28^. In this study, we found a novel mechanism underlying liver IRI. Similar to TLRs, which play a critical role in both infectious and sterile inflammation, we demonstrated that cytosolic RNA receptor RLRs and the downstream pathway that induces type I IFN production is also a significant inflammatory component for liver IRI, which had not been previously described. In addition, we found that the RNA-binding protein ADAR1 is required to prevent an excessive inflammatory reaction during IR. Although it was known that IFNs and their downstream pathway contribute to severe liver injury in the context of IRI^29^ how this signaling pathway was initially activated was not known. By studying the role of ADAR1 in hepatocytes, we identified a novel protective function for ADAR1: preventing aberrant sensing of endogenous RNA, even in the setting of non-infectious inflammatory conditions. We demonstrated that the cytosolic RNA-sensing signaling pathway is responsible for excessive type I IFN production, which was regulated by ADAR1. Suppression of ADAR1 significantly enhanced inflammation as well as liver damage following IR, which was accompanied by highly-expressed type I IFN in hepatocytes through cytosolic RLR-mediated RNA-sensing signaling pathways. In addition, primary hepatocytes also responded to other stressors such as endotoxin or dsRNA stimulation to activate this cytosolic RNA-sensing pathway, inducing type I IFN production and inflammatory reaction. These results indicate that the cytosolic RNA-sensing signaling pathway through RLRs regulated by ADAR1 plays a crucial role in liver IRI and is potentially also involved in other sterile tissue injuries.

ADAR1 is rapidly upregulated following ischemic stress and therefore, ADAR1 levels are likely to be tightly regulated to control the level of endogenous RNA-sensing through the RLR pathway. The liver is an active metabolic site and accordingly rapid RNA turn-over occurs in hepatocytes. Metabolism in hepatocytes changes dramatically when the cells are stressed and we observed that significantly-altered cytosolic RNA distribution patterns occurred during various stresses (data not shown). Cytosolic RNA-sensing RLRs are likely stimulated by endogenous RNAs to elicit stress responses and innate immune reactions. In the setting of liver IRI, this cytosolic pathway may serve as a potential target for liver protection at different levels of the pathway, such as at the very proximal receptor RLRs, adaptor protein IPS-1 and the downstream kinases TBK1 and IKK-ε. Interestingly, our results indicate that chemically inhibiting the pathway via BX-795 could significantly suppress IFNs, TNF-α, and IL-6 production and reduce H/R-induced cell damage. Thus, this novel signaling pathway may be a new drug target to prevent liver IRI or other stress-induced sterile organ injuries.

## Materials and Methods

### Preparation of ADAR1 Adenoviruses

Adenoviruses were prepared through a commercial service from Welgen, Inc. (Worcester, MA). Mouse ADAR1 cDNA (NM_001146296) and ADAR1 shRNA (sequence: GCC AAG AAC TAC TTC AAG AAA) were cloned into Pme1/Xho1 sites of a pENTCMV vector and recombined with the Ad5 backbone for virus preparation. Control viruses were also purchased from Welgen (scramble shRNA Cat# V1040and AdCMV empty Cat#V1000).

### Mice, virus transfection, and liver I/R model

The experimental protocols were approved by the Institutional Animal Care and Use Committee at the University of Pittsburgh and the Tongji Medical College (affiliated with the Huazhong University of Science and Technology). Wild type, C57BL/6 male mice (eight to 12 weeks old) were purchased from Jackson Laboratory.

The mice were treated with phosphate-buffered saline (PBS), control adenovirus, ADAR1 shRNA adenovirus, and ADAR1 cDNA adenovirus. A total of 5×10^10 ^PFU^30^ adenoviruses in 200 μl of PBS were administered via the tail vein 5 days before ischemia.

A partial (70%) hepatic IR mouse model^31^ was created under sodium ketamine (80 mg/kg, i.p.) and xylazine (10 mg/kg, ip) anesthesia. Following mid-line laparotomy, all structures in the portal triad to the left and median liver lobes were occluded with a micro-vascular clamp (Fine Science Tools) for 60 minutes and reperfusion was initiated by clamp removal. The temperature during ischemia was maintained at 32–33°C using a warming incubator chamber. After 6 hours of reperfusion, the mice were sacrificed. Blood and livers were collected for subsequent analysis. Plasma alanine aminotransferase (ALT) and aspartate transaminase (AST) levels were measured as indicators of liver damage using the DRI-CHEM 4000 Chemistry Analyzer System (Heska Loveland CO). The sham group received identical treatments without placement of the micro-vascular clamp.

### Histopathology

Formalin-fixed liver samples were embedded in paraffin and evaluated using hematoxylin and eosin (H&E) staining. All the pictures were captured on an Olympus Provis microscope. The necrotic area was assessed quantitatively using Image J software^10^,^32^ (National Institutes of Health, USA) as previously described. The ratio of necrotic area/whole area was normalized by the pathologist blinded to our experimental group.

### Hepatocyte isolation and cell culture

Hepatocytes were isolated from mice using a modified *in situ* collagenase (type H; Roche) perfusion technique^33^. Hepatocyte purity exceeded 99% and viability was typically greater than 85%, as determined by flow cytometric assay and trypan blue exclusion, respectively. Hepatocytes (150,000 cells) were plated onto gelatin-coated plates (Sigma) in Williams’ medium E with 10% calf serum, 15 mM HEPES, 2 mM L-glutamine, 100 units/ml penicillin, 100 units/ml streptomycin (Invitrogen), and10^−6 ^M insulin (Eli Lilly and Company). After 12 hours of incubation (37°C, 95% air-5% CO_2_), the medium was changed, and control adenovirus, ADAR1 shRNA adenovirus, or ADAR1 cDNA adenovirus at a multiplicity of infection (MOI) 1000 were added and incubated for 48 hours. After 48 hours, the medium was changed. Hepatocytes were exposed to hypoxia (1% O_2_) for 12 hours followed by 2 hours of reoxygenation (21% O_2_).

### Poly (I:C) treatment conditions

After treatment with different adenoviruses for 48 hours, the hepatocyte culture medium was replaced with medium containing 25 μg poly (I:C) (Sigma) for 8 hours for extracellular stimulation. For cytoplasmic poly (I:C) exposure, 50 μl of GeneJammer transfection reagent (Agilent Technologies Inc.)was mixed with 200 μl of Opti-MEM (Invitrogen) medium for 5 minutes at RT. This was followed by the addition of 25 μg poly(I:C) and incubation for 25 minutes at RT; the mixture was then added to the hepatocytes for 8 hours.

### Plasmid amplification

We used LyoComp GT116 for LucGL3 RIG-I or IPS-1 (InvivoGen) shRNA for transformation according to the manufacturer’s instructions. Cells were plated on LB plates with zeocin (InvivoGen) and after 16 hours of incubation at 37 °C, colonies were picked and amplified in LB medium with zeocin. The Endo Free Plasmid Maxi Kit (Qiagen) was used for plasmid isolation.

### RIG-I or IPS-1 co-transfection with adenovirus

GeneJammer transfection reagent (10 μl) was mixed with 200 μl of Opti-MEM medium for 5minutes at RT followed by the addition of 3 μg of control, RIG-I, or IPS-1 plasmid shRNA, incubated for 25 minutes at RT, and then added to the hepatocytes. After 8 to 10 hours of transfection, the medium was changed and MOI 1000 adenovirus was added to each well for 48 hours.

### BX795 treatment

A solution of 200 μM BX795 (InvivoGen), a TBK1/IKKε inhibitor, was added to hepatocytes eight hours before adenoviral transduction. The cells were treated with LPS or subjected to hypoxia/reoxygenation after 48 hours of transfection.

### Real-time PCR

Total RNA was isolated from hepatocytes using the RNeasy Mini Kit (Qiagen). cDNA was generated with 1 μg of total RNA and iScript reverse transcription supermix. Real-time PCR was subsequently performed using iTaq™ universal SYBR® Green supermixon a CFX96 real-time system(Bio-Rad) to analyze expression using 5′--3′ (forward [F]) and 5′--3′(reverse [R]) primers. iNOS: F: 5′-ACC AGA GGA CCC AGA GAC AA-3′, R: 5′-GCC TGG CCA GAT GTT CCT C-3′; IFN-β: F: 5′-TGA CGG AGA AGA TGC AGA AG-3′, R:5′-ACC CAG TGC TGG AGA AAT TG-3′; IFN-α F: 5′-CTA CTG GCC AAC CTG CTC TC-3′, R:5′- AGA CAG CCT TGC CAG GTC ATT-3′.RIG-I, F:AATCAGACAGATCCGAGACA; R:TGTCTTTC TCCAAAGCAAGT.ADAR1:F:CCGTACCATGTCCTGTAGTGACA; R:GCCCTTGGCTG AAAAGGTAAC. The TNF-α, IL-6, and IPS-1 primer were purchased from Qiagen. All data were normalized automatically using β-actin (Qiagen) as the loading control.

### Western blot

Total protein lysate was extracted from hepatocytes and liver tissue using lysis buffer (Cell Signaling Technology) and western blotting as previously described^6^. P38, phospho-P38, ERK, phospho-ERK, JNK, phospho-JNK, caspase 3, cleaved caspase 3, BCL-2, IRF1, and RIG-I were purchased from Cell Signaling Technology; ADAR1 antibody was purchased from Santa Cruz (cat# SC73408, clone 15.8.6). Secondary antibodies were purchased from Promega.

### Lactate dehydrogenase-release analysis, apoptosis testing, and cell viability assay

A total of 100 μl of the culture supernatant was removed from each well and added to a new plate to assay LDH activity (Promega) according to the manufacturer’s instructions. We further detected the rate of apoptosis using the PE Apoptosis Detection Kit I (BD) on the guava easyCyte^TM^ HT system and analysis on guavaSoft software (Millpore).The MTT kit was used for the hepatocyte viability assay according to the manufacturer’s instructions (Life Technologies).

### ELISA

TNF-α, IL-6, IL-10, and IFN-β levels were detected in culture medium or mouse plasma using ELISA (R&D) according to the manufacturer’s instructions.

### Statistical analysis

The data are expressed as means ± standard error (SEM) and analyzed with the statistical analysis software GraphPad Prism, Version 6.0 (GraphPad, San Diego, CA, USA). All data were analyzed using one-way analysis of variance (ANOVA) and Two-tail student’s t-test. when appropriate. Statistical significance was considered when P < 0.05.

## Additional Information

**How to cite this article**: Wang, H. *et al.* ADAR1 Suppresses the Activation of Cytosolic RNA-Sensing Signaling Pathways to Protect the Liver from Ischemia/Reperfusion Injury. *Sci. Rep.* **6**, 20248; doi: 10.1038/srep20248 (2016).
